# Modeling the Mechanism of GR/c-Jun/Erg Crosstalk in Apoptosis of Acute Lymphoblastic Leukemia

**DOI:** 10.3389/fphys.2012.00410

**Published:** 2012-11-19

**Authors:** Daphne Wei-Chen Chen, Marija Krstic-Demonacos, Jean-Marc Schwartz

**Affiliations:** ^1^Faculty of Life Sciences, University of ManchesterManchester, UK; ^2^Faculty of Life Sciences, Manchester Institute of Biotechnology, University of ManchesterManchester, UK

**Keywords:** glucocorticoid receptor, gene expression, kinetic simulation, systems biology, dynamic model

## Abstract

Acute lymphoblastic leukemia (ALL) is one of the most common forms of malignancy that occurs in lymphoid progenitor cells, particularly in children. Synthetic steroid hormones glucocorticoids (GCs) are widely used as part of the ALL treatment regimens due to their apoptotic function, but their use also brings about various side effects and drug resistance. The identification of the molecular differences between the GCs responsive and resistant cells therefore are essential to decipher such complexity and can be used to improve therapy. However, the emerging picture is complicated as the activities of genes and proteins involved are controlled by multiple factors. By adopting the systems biology framework to address this issue, we here integrated the available knowledge together with experimental data by building a series of mathematical models. This rationale enabled us to unravel molecular interactions involving c-Jun in GC induced apoptosis and identify Ets-related gene (Erg) as potential biomarker of GC resistance. The results revealed an alternative possible mechanism where c-Jun may be an indirect GR target that is controlled via an upstream repressor protein. The models also highlight the importance of Erg for GR function, particularly in GC sensitive C7 cells where Erg directly regulates GR in agreement with our previous experimental results. Our models describe potential GR-controlled molecular mechanisms of c-Jun/Bim and Erg regulation. We also demonstrate the importance of using a systematic approach to translate human disease processes into computational models in order to derive information-driven new hypotheses.

## Introduction

Acute lymphoblastic leukemia (ALL) refers to a cancer of T- or B-lymphoid progenitor cells, which is found to be the most common childhood malignancy (Pui et al., 2008). Despite the 85–90% cure rate in children (Onciu, 2009), a quarter of the cases suffer relapse, with drug resistance being a major cause (Mullighan et al., 2011). Glucocorticoids (GC) have been used as part of the treatment of many diseases including ALL, owing to their anti-inflammatory and anti-cancerous actions (Schaaf and Cidlowski, 2002). One of the main causes for resistance to GC is the defective signaling of GC to target genes in relation to apoptosis.

The principle of GC therapy in ALL is GC induced apoptosis, whereby GC activates the glucocorticoid receptor (GR) that upon hormone binding translocates to the nucleus and targets the apoptosis mediating family, the B-cell lymphoma 2 (Bcl-2). The Bcl-2 member Bim is known to be an essential initiator of apoptosis (Wang et al., 2003; Abrams et al., 2004; Ploner et al., 2007; Zhao et al., 2011) and an indirect GR target (Wang et al., 2003). The GR regulation of Bim in ALL is however not fully defined; it was reported that c-Jun may be a potential candidate for targeting Bim activation (Zhou and Thompson, 1996; Lu et al., 2006; Biswas et al., 2007; Chen et al., 2012). Apart from the Bcl-2 family members, we and others recently reported that the Ets protein, Ets-related gene (Erg) is induced by GC in the resistant ALL CEM C1–15 cells and may be a crucial GR target for determining GC resistance (Geng and Vedeckis, 2005; Baldus et al., 2006; Thoms et al., 2011; Tsuzuki et al., 2011).

Although recent high throughput technologies have advanced the understanding of complex gene regulatory mechanisms, it is important to note that complex molecular mechanisms cannot be deciphered using experimental data alone. Considering the wide range and large volume of presented data and information about GR, Bim, and Erg, computational modeling can be considered as an effective strategy for the interpretation of such data from various sources (Hoffmann et al., 2002; Faratian et al., 2009; Chen et al., 2010). In addition, modeling using time-course data not only raises the prospect of inferring the existence of causal relationships between genes, but also of identifying the direction of causality from the regulated genes (Sayyed-Ahmad et al., 2007). Among various modeling approaches, ordinary differential equations (ODE) have been widely used for studying the dynamics of gene networks. They offer the advantages of maintaining the quantitativeness and causality inherent in dynamical systems equations while being computationally manageable for small systems.

Recently we have proposed a series of ODE kinetic models for GR regulation by integrating time series of gene and protein expression data with kinetic modeling through information theory (Chen et al., 2010). We identified crucial time points that distinguish early GC and delayed GC response genes. To develop a more global understanding of GR action, we have extended this investigation to new time points and examined time-dependent GR-regulated genes with gene expression microarray. Time-course gene expression clustering led to further identification of crucial genes c-Jun and Erg as potential biomarkers for GC resistance (Chen et al., 2012).

**Figure A1:**
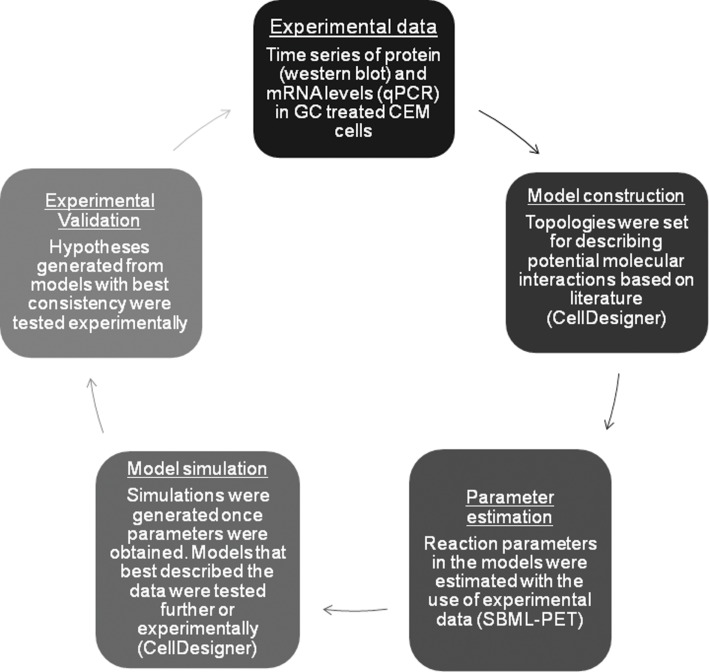
**Flow chart summarizing the workflow described in the introduction**.

Here we present extended models of GR regulation of c-Jun, Bim, and Erg based on a set of ODE (Chen et al., 2010). Several possibilities for interactions between GR and the selected genes were analyzed and the models that led to the best agreement with the experimental response were identified. We sought to show how our models can be further adapted to integrate and study GC regulated gene expression time series and obtain better understanding toward the regulatory mechanisms between GR, c-Jun, Bim, and Erg in leukemia (Figure A1 in Appendix).

## Materials and Methods

### Protein and mRNA expression measurements

Time-course protein and mRNA measurements were performed according to the methods described previously (Chen et al., 2010). In brief, CCRF-CEM-C1-15 ALL cells resistant to GCs and CCRF-CEM-C7-14 ALL cells sensitive to GCs were plated in six-well plates in RPMI-1640 supplemented with 10% DCC-FBS and incubated overnight. 1 μM of dexamethasone (Sigma, MO, USA), 10 μM YK-4-279, or 10 μM JNK inhibitor II (SP600125) were added to the medium and cells were incubated for 0, 2, 10, and 48 hours (h) accordingly. The relative protein expression was then measured and calculated via immuno-blotting using the ImageJ software, with actin as a control due to its ubiquitous expression in eukaryotic cells and its known property of being independent of GC treatment (Davies et al., 2008; Adzic et al., 2009). GR (H-300), c-Jun (H79), and Erg (D-3) antibodies were purchased from Santa Cruz Biotechnology (Santa Cruz, CA, USA); Actin and Bim antibodies were purchased from Abcam (Cambridge, UK). A detailed protocol and list of chemicals can be found in (Chen et al., 2010, 2012). The relative mRNA levels were measured using quantitative real time PCR analysis with the Bio-Rad Chromo4 system (Opticon monitor 3 software version 3.1) using the standard curve method with a known housekeeping gene RPL-19 as the control, which is not affected by GC (Rogatsky et al., 2003; Park et al., 2007; list of primer sequences: Rpl-19: F: ATGTATCACAGCCTGTACCTG; R: TTCTTGGTCTCTTCCTCCTTG; Bim: F: GAGAAGGTAGACAATTGCAG; R: GACAATGTAACGTAACAGTCG; GR: F: GTTGCTCCCTCTCGCCCTCATTC; R: CTCTTACCCTCTTTCTGTTTCTA; c-Jun: F: ACTGCAAAGATGGAAACGAC; R: AAAATGTTTGCAACTGCTGC; Erg: F: CAATCTCGAGCTATGGCCAGCACTATTAAGGAAGC; R: CAATCCCGGGTTAGTAGTAAGTGCCCAGATGAGAAG). Briefly, the total RNA was extracted from approximately 5 × 10^6^ cells with the RNeasy plus mini kit (Qiagen) and was used to generate cDNA using the Reverse-iT RTase kit following the two-step protocol (ABgene) according to the manufacturer’s guidelines. The cDNA was serially diluted and used for qPCR with SensiMix SYBR No-ROX Kit (Bioline) and the indicated primers. Thermocycle conditions were set with the Bio-Rad Chromo4 system (Opticon monitor 3 software version), including initial denaturation at 95°C (10 min), followed by 35 cycles at 95°C (30 s), 50°C (30 s) and 72°C (1 min). A melting curve was calculated from 72°C to 95°C with 1°C increase every 30 s. Standard curves were generated by plotting the CT vs. the $\log_{10}^{\text{copies}}$ of serially diluted PCR products in order to determine the copy number of amplified DNA. All results are reported as mean ± SD unless otherwise noted. The Tukey’s multiple comparison test and Student *t*-test were carried out to analyze western and qRT-PCR using SPSS 16.0 (SPSS Statistics).

### Signaling network representation

To construct the GR/c-Jun/Bim and GR/Erg pathways, literature information was used to assemble the signaling topologies. As described previously and in (Lu et al., 2006), GR activates Bim through an indirect mechanism in C7 cells, potentially through either direct or indirect c-Jun activation. Two models were built to represent GR/c-Jun/Bim in C7 cells, which differ by the involvement of an additional set of unknown protein X synthesis (Models 1 and 2). We have previously identified an increase in Erg expression in GC resistant C1 cells in response to GC treatment, and this was not found in GC sensitive C7 cells. In C1 cells, GR/Erg models were defined as Erg being either a direct or an indirect GR target (Models 3 and 4); a potential GC response element was identified via the champion ChiP transcription factor search portal which is a text mining tool based on SABiosciences’ database Decipherment of DNA element (DEDCODE). In C7 cells, although there were no significant changes in Erg expression, we did find a transient Erg recruitment on the GR promoter after 2 h of GC treatment. This highlights the potential role of Erg in regulating GR transcription. For this reason we constructed two models, one including only GR autoregulation in C7 cells (Ramdas et al., 1999; Schmidt et al., 2006), the other including both GR autoregulation and Erg regulation of GR transcription (Models 5 and 6). Therefore, we present six models, including two models representing GR regulation on c-Jun and Bim in C7 cells, two models for the Erg control of GR autoregulation in C7 cells and two models showing Erg being either a direct or indirect GR target in C1 cells. The network models were implemented using the CellDesigner software (www.celldesigner.org; Funahashi et al., 2003, 2008). Protein and mRNA degradation and basal synthesis rates were included in all models using mass action kinetics, without taking cellular translocation into consideration.

### Parameter estimation and simulation

All parameter estimations were performed using the Systems Biology Markup Language-based parameter estimation tool (SBML-PET; Zi and Klipp, 2006), which relies on the sets of ODE associated to model reactions and on the obtained experimental data. As shown in (Pedersen et al., 2004), GR has a relatively slow protein half-life between 27–42 h in the absence or presence of Dex, with a kinetic parameter of 0.0165–0.0257 h^−1^ (parameter *k* = ln(2)/protein half-life). For this reason, parameters were constrained between 0.01 and 1 for the estimation process. Once the estimated parameters were obtained, model simulations were then carried out using the CellDesigner software. Least-square residual values (ε) were calculated as seen in Chen et al., 2010 in order to determine the quality of the fit between simulations and experimental data.

$$\varepsilon \text{ = }\sqrt{\frac{1}{n}{\overset{n}{\underset{i\text{ = }1}{\sum }}\left(\frac{y_{\text{i}}-Y_{\text{i}}}{Y_{\text{i}}}\right)}^{2}}$$

where ε is the residual, *n* is the number of experimental data points, *y*_i_ are the experimental values, and *Y*_i_ are the simulated values of the variable under consideration.

## Results

### Modeling GR regulation of Bim via c-Jun activation in GC sensitive C7 cells

**Figure A2:**
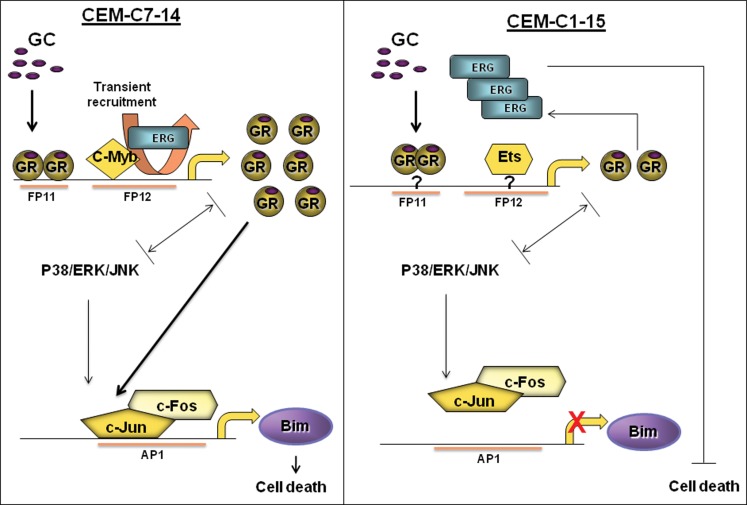
**Proposed regulation of cell death in leukemia**. In CEM C7 cells, upon GC treatment, GR becomes activated and alters Bim and GR transcription, potentially through AP-1 and Erg recruitment respectively. This may be cell specific as such recruitments were not seen in CEM C1 cells. Other factors such as MAPK signaling (i.e., JNK or P38) and c-myb may play a role in regulating GR, AP-1, and Bim.

GR induced apoptosis occurs through an intrinsic mitochondria dependent pathway via regulation of BCL-2 family proteins. In particular, the pro-apoptotic member Bim (BCL-2 interacting mediator of cell death) is known to be upregulated in sensitive ALL cells upon treatment with Dex through an indirect mechanism and plays a crucial role in apoptosis (Wang et al., 2003; Ploner et al., 2007; Chen et al., 2010). However, the molecular mechanism for Bim induction by GR is unclear. We and other have previously identified that c-Jun may be involved in the upregulation of Bim by GR (Zhou et al., 2000; Chen et al., 2010). Compared to the known direct GR target BclXL, both c-Jun and Bim were induced by GR much later, as a dramatic induction was observed between 6–10 h, suggesting a potential delayed and indirect GR induced activation mechanism (Chen et al., 2010). However, we cannot rule out the possibility that GR may induce c-Jun directly, as a putative GC response element was found at −1.6 kbp from the c-Jun promoter (Jonat et al., 1990). We also found two potential GR binding sites with the use of DECODE (Table A1 in Appendix). A schematic diagram of GR inducing c-Jun is presented in Figure A2 in Appendix.

**Figure 1:**
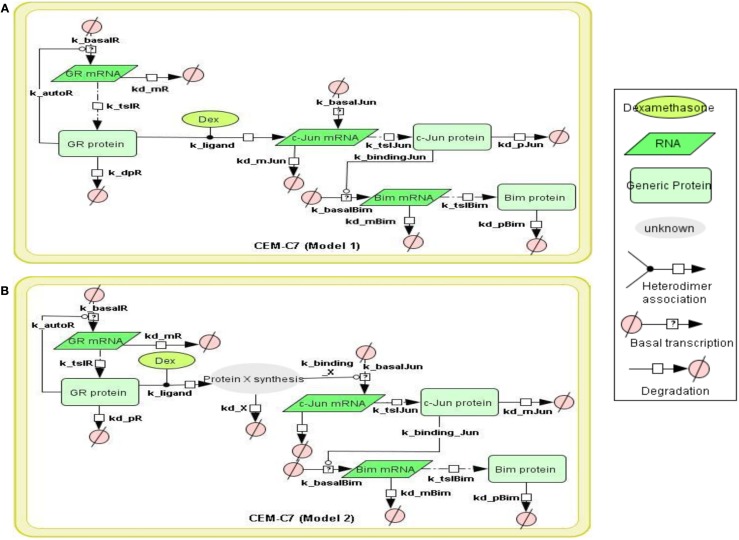
**Topology of GR/Jun/Bim models in CEM C7–14 cells Schematic representation of GR inducing Bim via c-Jun**. The figure summarizes the basic mechanism of Bim regulation controlled by GR. The model topology was based on (Chen et al., 2010, 2012), where glucocorticoid passes through the cell membrane, causes GR activation by dissociating GR from the cytoplasmic heat shock protein (HSP) complex. The bound GR dimerizes, either activates or represses its target genes through binding to GREs in the target genes or via the recruitment of other transcription factors. All models were constructed by CellDesigner, based on the known or potential molecular mechanisms but without taking the cytoplasmic-nuclear compartmentalization into account. Basal transcription, GR autoregulation, mRNA degradation, protein degradation, and binding dynamics were included in the models and the reactions were described using first order mass action kinetics. The details of the kinetic equations in all models are described in Appendix. **(A)** Model 1 represents GR induces Bim activation via direct binding to c-Jun. **(B)** Model 2 is similar to Model 1 but differs by the nature of the interaction between the GR and c-Jun. An extra step of protein synthesis was introduced for targeting down-stream target gene c-Jun in Model 2.

**Figure 2:**
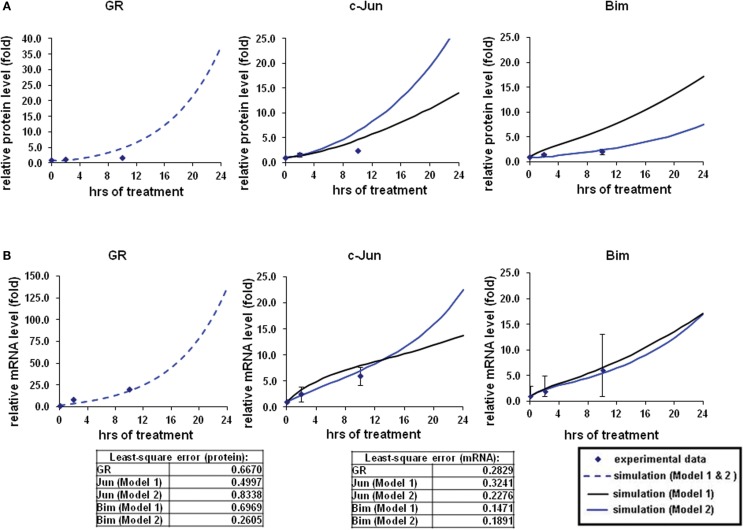
**Simulations of GR/Jun/Bim induction in CEM C7–14 cells**. The simulation process and the experimental data were described in our previous work (Chen et al., 2010, 2012). In brief, the expression dynamics were simulated with the use of CellDesigner and SBML-PET parameter estimation tool based on the experimental data obtained at 0, 2, and 10 h after 1 μM dexamethasone (Dex) treatment (Chen et al., 2012). Solid squares are the mean of the normalized experimental data and bars are the SDs for three sets of experiments. The simulation process is divided to two steps, the parameters for GR activation alone were first obtained (dotted line), and then the rest of the parameters were estimated based on the individual model topologies. The black solid line represents the simulation by Model 1 and the blue solid line is the simulation of Model 2 **(A)** Protein time-course simulations of GR, c-Jun, and Bim in CEM C7–14 cells. **(B)** The simulations for GR, c-Jun, and Bim mRNA dynamics. The models as shown revealed the characteristic kinetics of GR, c-Jun, and Bim in response with Dex in CEM C7–14 cells. The residual value was calculated to assess the quality of the fit between the simulations and the experimental data.

To model GR induced Bim activation, we considered two scenarios, involving either c-Jun being directly activated by GR before activating Bim (Model 1), or c-Jun being indirectly activated through *de novo* protein synthesis of an unknown protein X (Model 2; Figures 1A,B). The experimental data used for parameter simulation were taken from previous results (Chen et al., 2012), where protein and mRNA expression series were obtained after treatment with 1 μM Dex for 2 and 10 h in addition to the control (0 h). Model topologies were constructed via CellDesigner, with the inclusion of basal synthesis, protein, and mRNA degradation which were described by mass action kinetics; the equations used in the model are presented in Appendix. Cellular translocation and compartmental levels were not taken into account in the models. The unknown parameters were estimated from the defined topologies and the experimental data in combination with the use of SBML-PET. Due to the absence of quantitative data of most signaling components, we carried out the parameter estimation procedure in two parts: GR basal synthesis, protein, and mRNA degradation were estimated firstly, followed by the rest of unknown parameters. Figure 2 shows experimental and simulated time-courses using Models 1 and 2 of GR, c-Jun, and Bim protein and mRNA levels. All mRNA and proteins show induction but the simulation with Model 2 exhibited a more similar trend to our previous results (Chen et al., 2010, 2012), where a logarithmic tendency was seen. This observation is crucial since we have previously shown that direct and indirect GR targets can be distinguished based on the simulation trend, where direct GR targets exhibit a linear trend and indirect GR targets behave closer to a logarithmic function, due to the time delay required for the upstream pathway to be activated. At the protein level, Bim simulation seemed to fit better with Model 2, with an increase of up to 7.4-fold at 24 h and a smaller least-square residual value (ε = 0.2605) compared to Model 1 (17.18-fold at 24 h, ε = 0.6969; Figure 2A). In contrast, c-Jun protein simulation with Model 1 fits better (13.9-fold at 24 h, ε = 0.4997) than Model 2 (28.1-fold at 24 h, ε = 0.8338), particularly after 10 h of treatment (Model 1: 4.5-fold at 10 h; Model 2: 6.2-fold at 10 h). Compared to the simulations of protein levels, mRNA simulations with both Model 1 and Model 2 seemed to fit better with the experimental data (Figure 2B). A logarithmic shaped mRNA simulation of both Bim and c-Jun was seen with Model 2 only, in contrast an almost linear increase of Bim mRNA simulation was observed with Model 1. Least-square residual values in both models were much closer to each other for mRNA than proteins [Model 1: c-Jun (ε = 0.3241), Bim (ε = 0.1471); Model 2: c-Jun (ε = 0.2276), Bim (ε = 0.1891)].

### Modeling the role of Erg in GR gene expression in GC sensitive C7 cells

We have previously characterized the kinetic response to GR in ALL through time-course clustering of gene expression microarray (Chen et al., 2012). Results from the experiments and analyses identified Erg as one of the crucial genes that determine GC resistance. In addition, chromatin immunoprecipitation results showed that Erg was transiently recruited on the GR1A promoter in sensitive C7 cells only. Erg is a member of the Ets transcription factor family which has been linked to growth of hematopoietic cells and fusion with genes that are involved in cancer, such as the EWS gene in Ewing’s sarcoma (Sorensen et al., 1994; Tsuzuki et al., 2011). It has recently been identified as a prognosis factor in T-ALL and prostate cancer (Nam et al., 2007; Thoms et al., 2011; Tsuzuki et al., 2011). Ets subfamily members have also been linked to GR regulation in ALL (Geng and Vedeckis, 2005; Baldus et al., 2006; Figure A2 in Appendix).

**Figure 3:**
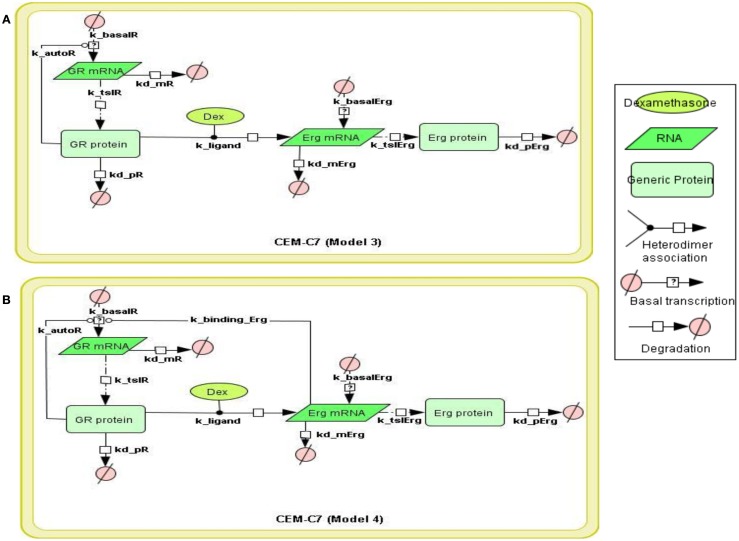
**Topology of GR/Erg models in CEM C7–14 cells**. Schematic representation of GR/Erg pathway. The nature of the topologies was based on previously established direct GR target model (Chen et al., 2010). Model 3 and Model 4 are similar and only differ by the regulation of GR, in Model 3 GR regulation is controlled by GR itself **(A)** whereas in Model 4 GR regulation is controlled by both GR itself and Erg direct interaction to GR gene **(B)**.

**Figure 4:**
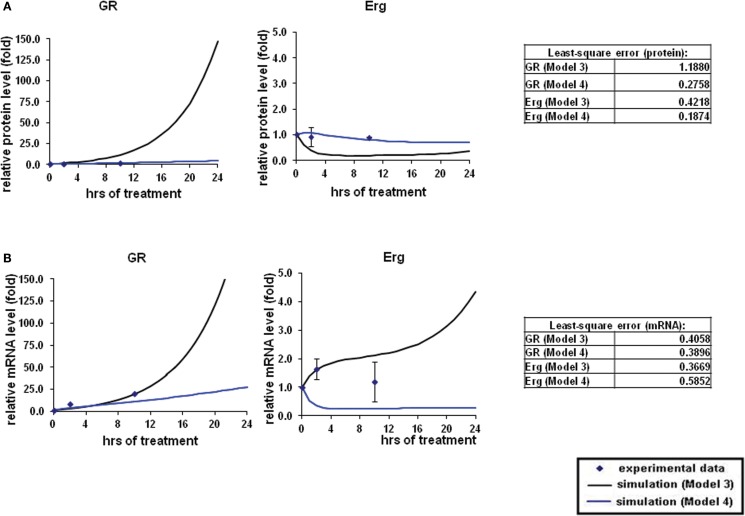
**Simulations of GR/Erg pathway in CEM C7–14 cells**. **(A)** Protein time-course simulation of GR/Erg pathway in CEM C7–14 cells. The same process of simulation was carried out in Model 3 and Model 4 as described in Figure 2. Solid squares are the experimental data and the error bars are means ± SD for three sets of experiments. The black solid line represents the simulation by Model 3 and the blue solid line is the simulation of Model 4. The residual value was calculated to assess the quality of the fit between the simulations and the experimental data. **(B)** GR and Erg mRNA time-course simulations in CEM C7–14 cells.

To systematically assess the factors of resistance to GC therapy in CEM cells, we developed kinetic models of GR/Erg signaling. As GR was recruited on the GR promoter in C7 cells, we sought to build a model that can capture the effect of Erg on GR expression and verify our experimental results. We here constructed two models, with Model 3 and Model 4 differing by Erg regulation on GR (Figures 3A,B). Both models captured GR and Erg transcription, translation, and degradation and each reaction was described by mass action kinetics (Appendix). The regulation between Erg and GR expression is not only an important component of GR homeostasis, but also a potential factor provoking GC resistance in relation to the level of GR. The simulations showed that Model 4 overall fits better our experimental results, where microarray, western, and qRT-PCR analysis identified a low level of Erg (≤1-fold) and an increase of GR (protein simulation increased up to 4.4-fold and mRNA up to 27.5-fold), although GR induction was smaller compared to GR simulations in Model 3 (with both protein and mRNA simulation exceeding 140-fold-change at 24 h). This is particularly apparent at the Erg mRNA level, in comparison with the low level of Erg mRNA simulation in Model 4, an increase was observed in Model 3 (4.3-fold at 24 h; Figures 4A,B). In addition, in Model 4 the simulation of both Erg protein and mRNA level decreased after 4 h of Dex treatment before gradually reaching a steady state (protein simulation: 0.9-fold, mRNA simulation 0.2-fold). At the protein level, least-square residual values for GR (ε = 1.1880) and Erg (ε = 0.4218) were both higher in Model 3 than in Model 4 (GR: ε = 0.2758; Erg: ε = 0.1874), with GR showing a greater discrepancy in comparison with the other components. The residual values of GR and Erg mRNA simulations were lower than their protein simulations in both Model 3 and Model 4, ranging between 0.3 and 0.5 (Model 3: GR: ε = 0.4058; Erg: ε = 0.3665; Model 4: GR: ε = 0.3896; Erg: ε = 0.5852). Altogether it seemed that Model 4 simulations and analysis corroborated earlier findings (Chen et al., 2012), which further confirms that this model is more appropriate. These results also highlight the importance of the role of Erg expression on GR regulation.

### Modeling the role of Erg in GR gene expression in GC resistant C1 cells

**Figure 5:**
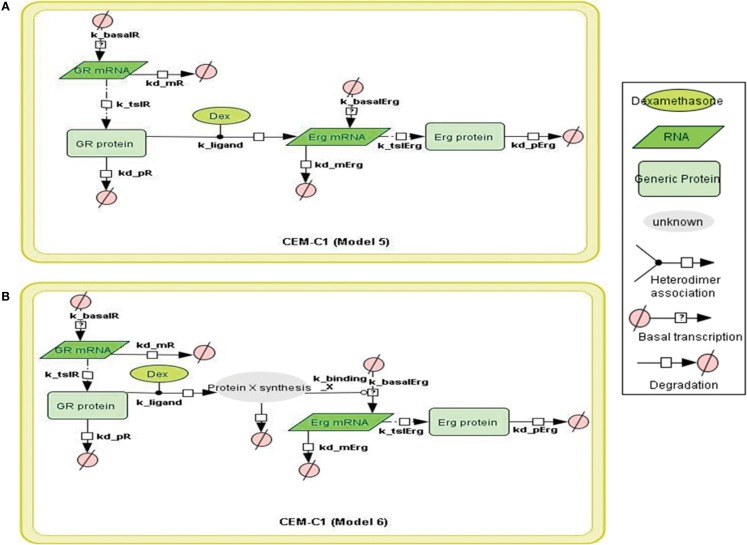
**Topology of GR/Erg models in CEM C1–15 cells**. Schematic representation of GR/Erg pathway in CEM-C1–15 cells. The nature of the topologies was based on previously established direct and indirect GR target models in CEM-C1–15 cells (Chen et al., 2010). GR autoregulation was not included in CEM-C1–15, Model 5 and Model 6 differ by the GR regulation on Erg induction, with Model 5 indicating Erg as a direct GR target **(A)** and Model 6 showing Erg as an indirect GR target where *de novo* protein synthesis is required for Erg induction **(B)**.

In the next step, we sought to investigate the GR dependent Erg induction that was previously found in resistant C1 cells only. Treatment with Dex was found to induce Erg expression in C1 cells and an increase of apoptosis was observed when treated with Erg inhibitor (Chen et al., 2012). GR may be able to directly activate Erg, as a potential GRE was identified on the Erg promoter (Table A1 in Appendix). Based on the experimental data obtained and strategy outlined above, we created two models for the GR induced Erg expression (Figure 5). Similar to the direct and indirect models as established in (Chen et al., 2010), the two models differed by an extra step of *de novo* protein synthesis (Figures 5A,B); the positive GR autoregulation feedback loop was not included in C1 cells (Ramdas et al., 1999; Schmidt et al., 2006; Chen et al., 2010).

**Figure 6:**
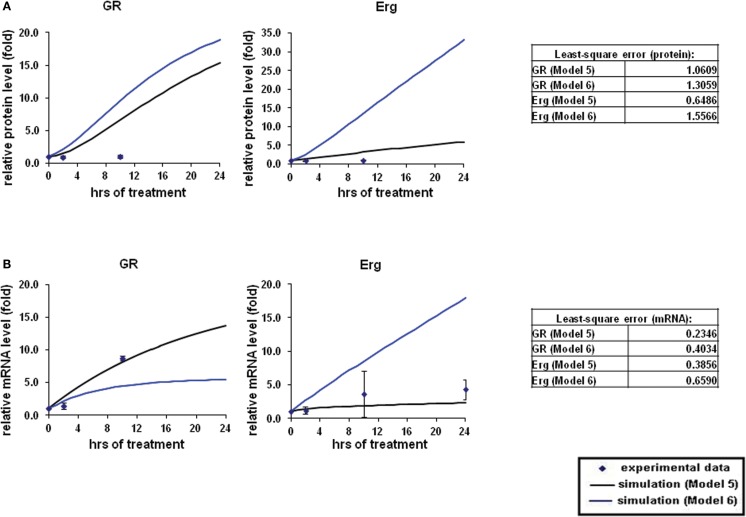
**Simulations of GR/Erg pathway in CEM C1–15 cells**. The same process of simulation was carried out in Model 5 and Model 6 as described in Figure 2. Solid squares are the experimental data and the error bars are means ± SD for three sets of experiments. The black solid line represents the simulation by Model 5 and the blue solid line is the simulation of Model 6. **(A)** Protein time-course simulation of GR/Erg pathway in CEM-C1–15 cells. **(B)** GR and Erg mRNA time-course simulations in CEM-C1–15 cells.

The overall simulations with both direct (Model 5) and indirect models (Model 6) showed an induction in both GR and Erg protein and mRNA levels (Figures 6A,B). At the protein level, GR protein simulations showed an increase of ≥15-fold with both models whereas protein simulation with Model 6 increased at a much lower rate in comparison (2.3-fold at 24 h) with Model 5. Erg protein simulation with Model 5 seemed to fit better than Model 6, with the simulation clearly showing a better fit to the experimental data. Despite the consistent upregulating simulation trends compared with the experimental results, least-square analysis showed that apart from Erg with Model 5 (ε = 0.6486), the residual values were all greater than 1 [Model 5: GR (ε = 1.5566) and Model 6: GR (ε = 1.0605), Erg (ε = 1.3059)]. In contrast to protein simulations, mRNA simulations were found to have a good fit to the experimental data with both models, where GR and Erg mRNA were both induced. The mRNA simulations with both models appeared to increase at a slower rate in comparison with the protein simulations; in Model 5 GR mRNA increased up to 13.6-fold and Erg mRNA up to 2.3-fold at 24 h, whereas Model 6 showed a lower increase rate with GR mRNA (5.4-fold) but a higher and more linear induction in Erg mRNA (17.9-fold). Much smaller residual values were calculated at the mRNA levels, with Model 5 (GR: ε = 0.2346, Erg: ε = 0.3856) having a better fit than Model 6 (GR: ε = 0.4034, Erg: ε = 0.6590). These findings support the hypothesis that GR may activate Erg expression by directly targeting the GRE on the Erg promoter.

## Discussion

Glucocorticoids play a pivotal role in the treatment of ALL through initiating apoptosis. Despite the relatively high cure rate, GC resistance still remains a therapeutic problem due to its unknown molecular mechanism. Thanks to advances in “omics” technologies, there is a growing amount of literature and molecular models addressing GC induced signaling. Bim, a well known pro-apoptotic Bcl-2 member, has been identified as a crucial player in apoptosis and is able to trigger cell death (Ploner et al., 2007). It is known that Bim is activated by GR via an indirect mechanism where *de novo* protein synthesis is required (Wang et al., 2003). Nevertheless, the exact mechanism of Bim induction by GR is poorly understood. Forkhead box subgroup O3a (Foxo3a) has been suggested as a potential candidate for targeting and activating Bim, thereby initiating apoptosis in chronic myeloid leukemia (Essafi et al., 2005). This however may not be the case in ALL as we observed an upregulation of Bim and GILZ in C7 cells, but not of Foxo3 (Chen et al., 2010, 2012). GILZ can protect ALL cells by provoking nuclear exclusion of Foxo3 (Latré de Laté et al., 2010), which may also occur in ALL and further supports the idea that an alternative protein is involved in Bim induction (Lu et al., 2006). Time-course microarrays in ALL (Chen et al., 2012) and a study of Bim in neuronal cells (Biswas et al., 2007) had led us to hypothesize that c-Jun may be a crucial player in Bim activation. On the other hand, Erg has recently been identified as a crucial prognosis factor for determining GC resistance (Tsuzuki et al., 2011). We have verified these results and found that Erg signaling may be involved in GR regulation with a cell specific mechanism (Chen et al., 2012).

**Figure A3:**
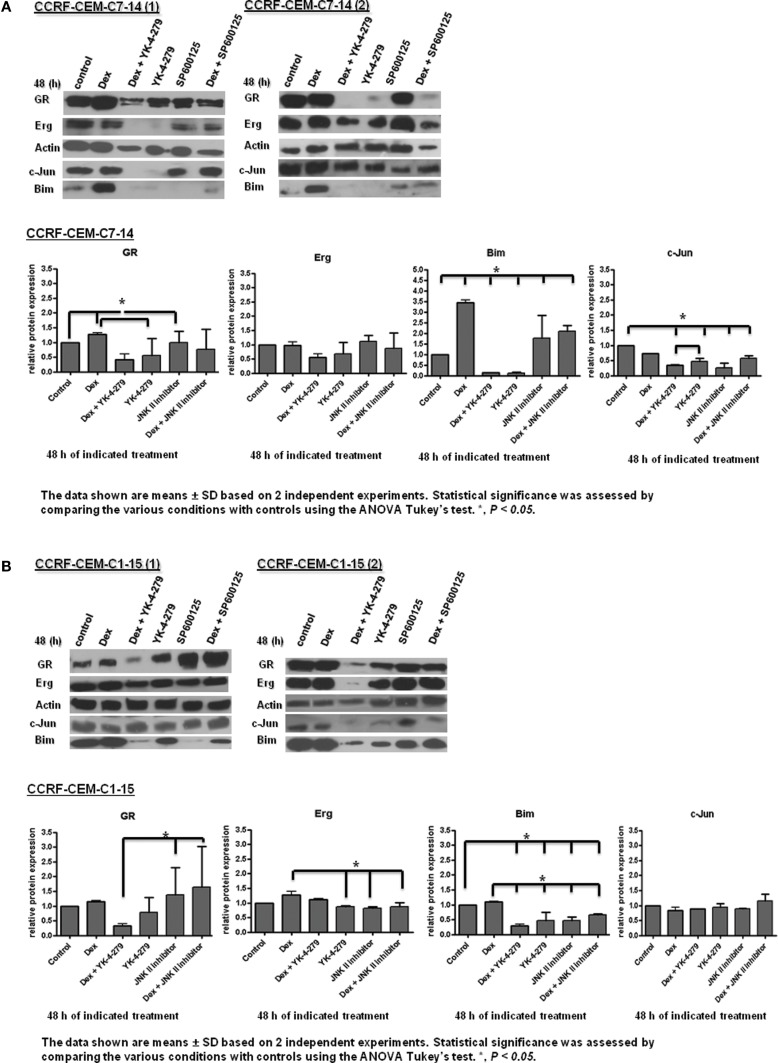
**GR target gene protein expression in CEM cells**. Western blot analysis of GR, Erg, c-Jun, and Bim protein levels, with actin as a control in C7 **(A)** and C1 **(B)** cells cultured with a combination of 1 μM Dex, 10 μM YK-4-279, 10 μM JNK inhibitor (SP600125) for 48 h. Protein levels were quantified by ImageJ, normalized to actin and presented as a histogram. Error bars represent ± SD of two independent experiments. An asterisk indicates a significant difference at *p* < 0.05 using ANOVA and Tukey’s test.

Our ODE models of GR induced apoptosis capture the dynamics of GR regulation of Bim via c-Jun and the crosstalk between GR and Erg. For GR/c-Jun/Bim regulation, two possible topologies were constructed, where the two models differed by the nature of the *de novo* protein synthesis (Figures 1A,B). Simulation outcomes of GR interaction with c-Jun and Bim were consistent with the available biological data, where GR induced c-Jun and Bim over time (Zhou and Thompson, 1996; Zhao et al., 2011). Least-square analysis showed that c-Jun mRNA and Bim protein in Model 2 fitted better with the experimental data whereas c-Jun protein and Bim mRNA fitted better with Model 1 (Figures 2A,B). These results may be explained by an involvement of alternative mechanisms, as treatment with the protein synthesis inhibitor cycloheximide has shown that c-Jun expression was not affected and the induction became more prominent. Further nuclear run on tests have indicated that c-Jun induction requires at least 6 h after Dex treatment. This process takes much longer than the time required for GR translocation and binding to the promoter of the target gene, as previous green fluorescent protein experimentation indicated that a full GR translocation takes only 2 h to complete (Htun et al., 1996). It was suggested that the mechanism for the delayed c-Jun induction may be secondary, potentially through the relief from a protein repressor of transcription (Zhou et al., 2000). Our previous chromatin immunoprecipitation results had revealed c-Jun recruitment on the Bim promoter, which was not repressed by inhibition of c-Jun N-terminal kinases (JNKs); in fact apoptosis was enhanced in C7 cells despite Bim expression being reduced (Figure A3A in Appendix; Lu et al., 2006; Chen et al., 2012), suggesting an alternative signaling pathway for c-Jun induction. Several potential mechanisms of GR-regulated Bim induction have been suggested, including the MAPK pathway (Morton et al., 2003; Lu et al., 2006; Dobreva et al., 2009; Rahim et al., 2011).

Another subject of special interest to this study was to investigate the role of Erg in determining GC resistance, since we identified Erg recruitment on the GR promoter in C7 cells only but a substantial Erg expression was found in resistant C1 cells (Chen et al., 2012). Since the relation between GR and Erg remains obscure, we aimed to evaluate the role of Erg in the GR signaling pathway, which could be modulated in a cell dependent manner. Our GR/Erg models were devised into four sets, with two potential mechanisms each tested in C7 and C1 cells.

To place Erg in the GR induction model in C7 cells, we considered the regulatory influence of Erg on GR autoregulation. Based on previous literature and the finding of Erg recruitment on the GR promoter, we hypothesized that Model 4 with direct Erg regulation on GR expression would show a better fit with the experimental data than Model 3 (Figures 3A,B; Geng and Vedeckis, 2005; Chen et al., 2012). Indeed, simulations with Model 4 showed an increase in GR mRNA and protein levels, and more importantly a low level of Erg protein and mRNA, despite that a more dramatic increase of GR protein and mRNA levels were identified in Model 3 simulations (Figures 4A,B). Preliminary western blotting based on two sets of independent experiments confirmed the potential role of Erg in GR regulation when treated with a functional inhibitor of Erg, YK-4-279. In this case, a much lower GR protein expression was identified in the presence and absence of Dex (Figure A3A in Appendix). The results showed that despite limited data, Model 4 was still able to reflect the expected experimental observations, where a low level of Erg was found in CEM-C7–14 cells, to a good extent. It should be noted that we also identified a depletion of Bim protein expression when treated with YK-4-279, which suggested a possible role for Erg in the regulation of Bim (Figure A3 in Appendix). The correlated apoptosis assay with annexin V, however, still showed an increase in apoptosis, indicating a potential switch to an alternative apoptotic signaling pathway. More investigation is required to clarify these results (Chen et al., 2012). Yu et al. (2010) conducted a detailed study on Erg in the GR subfamily-androgen receptor (AR) signaling and have shown that Erg may inhibit AR by a negative autoregulatory loop, or by Erg affecting the AR target gene selectivity (Chen and Sawyers, 2010). Since AR and GR share high homology in the DNA binding domain and recognize similar hormone response elements, the abovementioned mechanism should be considered in the GR/Erg model (Forman and Samuels, 1990; Laudet et al., 1992).

In the next step, we aimed to determine whether Erg acts as a direct GR target, as the positive regulation of Erg by GR was only observed in the resistant C1 cells and a consensus GRE was identified in Erg. By adapting the direct GR and indirect GR model in C1 cells as indicated in (Chen et al., 2010), two models were created (Figure 5). GR autoregulation was not considered in resistant C1 cells and the two models only differed by a step of *de novo* protein X synthesis. The simulation results showed an induction of GR and Erg protein and mRNA levels with both Model 5 and Model 6, with GR protein and Erg mRNA and protein showing a more dramatic increase in Model 6 (Figures 6A,B). By observing the trend and evaluating the residual values, which appeared to fit better with Model 5 in all cases, we hypothesize that Erg is more likely to be a direct GR target. Further experimental results are required to test this prediction. Western blotting of Dex in combination with YK-4-279 treatment showed a lower Bim expression than the control in C1 cells (Figure A3B in Appendix), even though a significant increase in apoptosis was identified previously (Chen et al., 2012). This suggests that Erg may either be acting as an activator upstream of anti-apoptotic target genes, or as a repressor of pro-apoptotic signaling but not via Bim activation.

We have built quantitative models to study c-Jun, Bim, and Erg signaling and their interactions with GR. Although ODE models are unable to account for cell-to-cell variability, they are sufficient to describe and test the major dynamical trends of signaling pathway components and their interactions. Our models account for established as well as novel experimental observations regarding the interplay between GR, c-Jun, and Bim (Model 1 and 2), and point out how Erg is regulated as a cell specific modulator (Model 3 and 6). Taking the experimental observations into account, this systems biology approach allowed us to differentiate between alternative mechanisms and determine the likely roles of c-Jun and Erg in the network. Our models can serve as a basis to study GR/c-Jun/Bim and GR/Erg signaling in ALL and can be continuously extended as more data become available. Overall, this study shows that a systems biology approach combining mechanistic modeling with experimental analysis is of valuable help to dissect complex signaling pathways and improve our understanding toward disease and drug action. As modeling allows us to test alternative mechanisms and identify more likely scenarios, experimental priorities can be defined to test the newly generated hypotheses and data sets can be expanded to increase the computational precision. In the present study, our models not only reflect the Erg/GR regulation in C7 cells but also help defining future experimental priorities, which include: the identification of potential regulatory mechanisms that mediate c-Jun transcription through the analysis of the c-Jun promoter for a potential GC sensitive repressor; the use of cycloheximide and chromatin immunoprecipitation to examine the interaction between Erg and GR in C1 cells.

## Conflict of Interest Statement

The authors declare that the research was conducted in the absence of any commercial or financial relationships that could be construed as a potential conflict of interest.

## Appendix 1

### Kinetic equations describing GR mediated induction of Bim, c-Jun, and Erg

The set of ordinary differential equations describes the GR regulatory kinetics implemented in our models. Here, the kinetics is essentially the same but with different protein or mRNA names. *kd_X* represents the overall degradation of factor X; *k_binding_X* is the regulation between the unknown *proteinX* and the target gene; *k_binding_Erg* is the regulation between Erg and GR; *k_ligand* is the rate of complex association of dexamethasone and GR; *kd_m* and *kd_p* represent the first order rate constants of the degradation of mRNA and protein respectively. The term *Tsl* denotes translation, *basal* denotes basal transcription, *proteinX* the unknown protein, and *R* the glucocorticoid receptor.

## Appendix 2

### Model 1 and 2

#### GR

$$\frac{d\left[R\text{\_mRNA}\right]}{dt}\text{ = }k\text{\_basal}R\text{ + [}R\text{]}\cdot k\text{\_auto}R-\left[R\text{\_mRNA}\right]\cdot k\text{d\_mR}$$

$$\frac{d\left[R\right]}{dt}\text{ = }\left[R\text{\_mRNA}\right]\cdot k\text{\_tsl}R-\text{[}R\text{]}\cdot k\text{d\_pR}$$

### Model 1

#### Bim

$$\frac{d\left[\text{Bim\_mRNA}\right]}{dt}\text{ = }k\text{\_basalBim + [Jun]}\cdot k\text{\_binding\_Jun}-\left[\text{Bim\_mRNA}\right]\cdot k\text{d\_mBim}$$

$$\frac{d\left[\text{Bim}\right]}{dt}\text{ = }\left[\text{Bim\_mRNA}\right]\cdot k\text{\_tslBim}-\text{[Bim]}\cdot k\text{d\_pBim}$$

#### Jun

$$\frac{d\left[\text{Jun\_mRNA}\right]}{dt}\text{ = }k\text{\_basalJun}-\text{[Jun\_mRNA]}\cdot k\text{d\_mJun + }\left[\text{Dex}\right]\cdot \left[R\right]k\text{\_ligand}$$

$$\frac{d\left[\text{Jun}\right]}{dt}\text{ = }\left[\text{Jun\_mRNA}\right]\cdot k\text{\_tslJun}-\text{[Jun]}\cdot k\text{d\_pJun}$$

### Model 2

#### Bim

$$\frac{d\left[\text{Bim\_mRNA}\right]}{dt}\text{ = }k\text{\_basalBim + [Jun]}\cdot k\text{\_binding\_Jun}-\left[\text{Bim\_mRNA}\right]\cdot k\text{d\_mBim}$$

$$\frac{d\left[\text{Bim}\right]}{dt}\text{ = }\left[\text{Bim\_mRNA}\right]\cdot k\text{\_tslBim}-\text{[Bim]}\cdot k\text{d\_pBim}$$

#### Jun

$$\frac{d\left[\text{Jun\_mRNA}\right]}{dt}\text{ = }k\text{\_basalJun + [ProteinX\_synthesis]}\cdot k\text{\_binding\_X}-\left[\text{Jun\_mRNA}\right]\cdot k\text{d\_mJun}$$

$$\frac{d\left[\text{Jun}\right]}{dt}\text{ = }\left[\text{Jun\_mRNA}\right]\cdot k\text{\_tslJun}-\text{[Jun]}\cdot k\text{d\_pJun}$$

### Protein X synthesis

$$\frac{d\left[\text{ProteinX\_synthesis}\right]}{dt}\text{ = }\left[\text{Dex}\right]\cdot \left[R\right]\cdot k\text{\_ligand}-\text{[ProteinX\_synthesis]}\cdot k\text{d\_X}$$

### Model 3

#### GR

$$\frac{d\left[R\text{\_mRNA}\right]}{dt}\text{ = }k\text{\_basal}R\text{ + [}R\text{]}\cdot k\text{\_auto}R-\left[R\text{\_mRNA}\right]\cdot k\text{d\_mR}$$

$$\frac{d\left[R\right]}{dt}\text{ = }\left[R\text{\_mRNA}\right]\cdot k\text{\_tsl}R-\text{[}R\text{]}\cdot k\text{d\_pR}$$

#### Erg

$$\frac{d\left[\text{Erg\_mRNA}\right]}{dt}\text{ = }k\text{\_basalErg}-\left[\text{Erg\_mRNA}\right]\cdot k\text{d}\text{\_mErg + [}R\text{]}\cdot \left[\text{Dex}\right]\cdot k\text{\_}\text{ligand}$$

$$\frac{d\left[\text{Erg}\right]}{dt}\text{ = }\left[\text{Erg\_mRNA}\right]\cdot k\text{\_tslErg}-\text{[Erg]}\cdot k\text{d\_pErg}$$

### Model 4

#### GR

$$\frac{d\left[R\text{\_mRNA}\right]}{dt}\text{ = }k\text{\_basal}R\text{ + [}R\text{]}\cdot k\text{\_auto}R-\left[R\text{\_mRNA}\right]\cdot kd\text{\_m}R\text{ + [Erg\_mRNA]}\cdot k\text{\_}\text{binding}\text{\_}\text{Erg}$$

$$\frac{d\left[R\right]}{dt}=\left[R\text{\_mRNA}\right]\cdot k\text{\_}\text{tsl}R-\left[R\right]\cdot k\text{d\_pR}$$

#### Erg

$$\frac{d\left[\text{Erg\_mRNA}\right]}{dt}\text{ = }k\text{\_basalErg}-\text{[Erg\_mRNA]}\cdot k\text{\_dmErg + [}R\text{]}\cdot \left[\text{Dex}\right]\cdot k\text{\_}\text{ligand}$$

$$\frac{d\left[\text{Erg}\right]}{dt}\text{ = [Erg\_mRNA]}\cdot k\text{\_tslErg}-\text{[Erg]}\cdot k\text{d\_pErg}$$

### Model 5

#### GR

$$\frac{d\left[R\text{\_mRNA}\right]}{dt}\text{ = }k\text{\_}\text{basal}R-\text{[}R\text{\_mRNA]}\cdot k\text{d\_mR}$$

$$\frac{d\left[R\right]}{dt}\text{ = [}R\text{\_mRNA]}\cdot k\text{\_tsl}R-\text{[}R\text{]}\cdot k\text{d\_pR}$$

#### Erg

$$\frac{d\left[\text{Erg\_mRNA}\right]}{dt}\text{ = }k\text{\_basalErg}-\text{[Erg\_mRNA]}\cdot k\text{d\_mErg + [}R\text{]}\cdot \left[\text{Dex}\right]\cdot k\text{\_}\text{ligand}$$

$$\frac{d\left[\text{Erg}\right]}{dt}\text{ = [Erg\_mRNA]}\cdot k\text{\_tslErg}-\text{[Erg]}\cdot k\text{d\_pErg}$$

### Model 6

#### GR

$$\frac{d\left[R\text{\_mRNA}\right]}{dt}\text{ = }k\text{\_}\text{basal}R-\text{[}R\text{\_mRNA]}\cdot k\text{d\_mR}$$

$$\frac{d\left[R\right]}{dt}\text{ = [}R\text{\_mRNA]}\cdot k\text{\_tsl}R-\text{[}R\text{]}\cdot k\text{d\_pR}$$

#### Erg

$$\frac{d\left[\text{Erg\_mRNA}\right]}{dt}\text{ = }k\text{\_basalErg}-\text{[Erg\_mRNA]}\cdot k\text{\_dmErg + [ProteinX\_synthesis]}\cdot k\text{\_}\text{binding}\text{\_}\text{X}$$

$$\frac{d\left[\text{Erg}\right]}{dt}\text{ = [Erg\_mRNA]}\cdot k\text{\_tslErg}-\text{[Erg]}\cdot k\text{d\_pErg}$$

### Protein X synthesis

$$\frac{d\left[\text{ProteinX\_synthesis}\right]}{dt}\text{ = [Dex]}\cdot \left[R\right]\cdot k\text{\_ligand}-\text{[ProteinX\_synthesis]}\cdot k\text{d\_X}$$

**Table A1:** **Location and sequence of binding sites in the regulatory regions of the indicated genes**.

Transcription factor	Binding protein	Strand	Sequence
**A. LOCATION AND SEQUENCE OF POTENTIAL GR BINDING SITES IN C-JUN**			
GR	Chr1:59247433–59247449	+	AGGTCCATGCAGTTCTT
GR	Chr1:59247576–59247595	–	TCGTGCACACTGGGGGCGCC
**B. LOCATION AND SEQUENCE OF POTENTIAL GR BINDING SITES IN ERG**			
GR	Chr1:40036930–40036946	–	CAATAACACGTGGTGAC

*Potential GRE sites were identified using the text mining tool – The Champion ChiP Transcription Factor Search Portal (CCTSF) from Qiagen SABiosciences’s database. (A) Location and sequence of potential GR binding sites in c-Jun. (B) Location and sequence of GR binding sites in Erg*.

## Abbreviations

AP-1, activator protein 1; Bcl-2, B-cell lymphoma 2; Dex, dexamethasone; Erg, Ets-related gene; GC, glucocorticoid; GR, glucocorticoid receptor; GRE, glucocorticoid response element; ODE, ordinary differential equations.
